# The ICECAP-A instrument for capabilities: assessment of construct validity and test–retest reliability in a general Dutch population

**DOI:** 10.1007/s11136-021-02980-5

**Published:** 2021-08-31

**Authors:** Pieter J. Rohrbach, Alexandra E. Dingemans, Brigitte A. Essers, Eric F. Van Furth, Philip Spinhoven, Catharina G. M. Groothuis-Oudshoorn, Janine A. Van Til, M. Elske Van den Akker-Van Marle

**Affiliations:** 1 Department of Psychiatry, Leiden University Medical Center, Leiden, The Netherlands; 2 GGZ Rivierduinen, Eating Disorders Ursula, Leiden, The Netherlands; 3 Department of Clinical Epidemiology and Medical Technology Assessment, Maastricht University Medical Centre, Maastricht, The Netherlands; 4 Institute of Psychology, Leiden University, Leiden, The Netherlands; 5 Department of Health Technology and Services Research, Technical Medical Centre, University of Twente, Enschede, The Netherlands; 6 Department of Biomedical Data Sciences, Section Medical Decision Making, Leiden University Medical Center, P.O. Box 9600, 2300 RC Leiden, The Netherlands

**Keywords:** Capabilities, Well-being, Psychometric assessment, Known-group validity

## Abstract

**Purpose:**

The ICEpop CAPability measure for Adults (ICECAP-A) assesses five capabilities that are important to one’s well-being. The instrument might be an important addition to generic health questionnaires when evaluating quality of life extending beyond health. This study aimed to conduct a psychometric assessment of the Dutch translation of the ICECAP-A.

**Methods:**

Construct validity of the instrument was assessed in two ways. First, by measuring correlations with the EQ-5D-5L questionnaire and a measure of self-efficacy and, second, by investigating the ability to distinguish between groups known to differ on the construct the ICECAP-A means to capture. Additionally, test–retest reliability was evaluated.

**Results:**

In total, 1002 participants representative of the general Dutch population completed an online survey. For test–retest reliability, 252 participants completed the same questionnaire 2 weeks later. The ICECAP-A indicated moderate to strong correlations with the EQ-5D-5L and a strong correlation with self-efficacy. Furthermore, it was capable of differentiating known groups. Moreover, results indicated adequate test–retest reliability with an intraclass correlation coefficient of 0.79.

**Conclusion:**

In summary, results suggest adequate test–retest reliability and construct validity and indicate that the ICECAP-A might be of added value, especially when considering areas outside of the traditional health intervention model.

**Supplementary information:**

The online version contains supplementary material available at 10.1007/s11136-021-02980-5.

## Plain English summary

It is important to be able to precisely measure quality of life, because that helps in assessing how effective a treatment is. The ICEpop CAPability measure for Adults (ICECAP-A) is a questionnaire that was developed to capture one’s quality of life in terms of general well-being. This study aimed to further clarify what the ICECAP-A exactly measures and whether it can do so reliably. That would help to decide when this questionnaire should be used. The main finding of the study is that the ICECAP-A questionnaire indeed captures a concept (related to, but different from physical health) best described as well-being. It does so in a valid and reliable way. This suggests that the ICECAP-A questionnaire can be used to measure quality of life. It will be especially useful in contexts outside the area physical health, such as public health, social care, chronic illness, and mental health.

## Introduction

Generic health questionnaires are often used to measure benefits of interventions, even in situations where relevant improvements might not be captured in terms of health. As such, they are criticized to employ a narrow view on quality of life, with emphasis on physical aspects of health and current functional abilities rather than resources, coping capabilities, and general well-being [1–4]. Certain aspects of quality of life that fall beyond physical health might be underestimated, such as living situations, social support systems, psychological resilience, and the capability to cope with illness. Consequently, this can lead to an undervaluation of effect when assessing the benefits of an intervention, especially in the context of social care, mental health [5, 6], public health, general well-being, chronic illness, and elderly care. The ICEpop CAPability measure for Adults (ICECAP-A) [7] assesses one’s quality of life in terms of capabilities and might be better suited than generic health questionnaires in cases that do not fit the traditional health intervention model. Establishing the reliability and validity of the ICECAP-A is vital in order to confidently use this instrument in studies as a complement to generic health questionnaires (i.e., when changes or improvement in outcomes beyond health alone are expected).

Afentou and Kinghorn [8] have systematically reviewed the literature for studies exploring the psychometric properties of the ICECAP-A. Included studies suggested the ICECAP-A to be positively correlated with concepts such as feelings of happiness and freedom [9] and moderately or strongly related to health-related quality of life instruments [10, 11]. Helter et al. [12] found similar results concerning the psychometric qualities of the ICECAP-A in a more general systematic review on the use of capability instruments in economic evaluations. Overall, the evidence suggests adequate content and construct validity of the ICECAP-A. Its construct seems to be related to quality of life as measured by generic health questionnaires, albeit conceptually different [8]. Few studies have investigated the test–retest reliability of the ICECAP-A [13, 14], so more information on this parameter is required. Additionally, the majority of studies assessing the psychometric properties were conducted in the UK [8], the results of which do not necessarily generalize to translations of the instrument and other countries. At the moment, nine translations of the ICECAP-A exist (i.e., Chinese, Danish, Dutch, French, German, Hungarian, Italian, Persian, and Welsh) and an increasing number of studies is available on the psychometric properties of these translations [14–19]. Assessing the psychometric properties of translations of the ICECAP-A in other countries not only makes it more widely available, but strengthens the confidence in the instrument as a whole. To our knowledge there have been no attempts to assess the psychometric properties of the Dutch translation of the ICECAP-A beyond its face validity [20]. The current aim of the study is to assess the test–retest reliability and improve the understanding of the construct validity of the Dutch translation of the ICECAP-A.

## Methods

### Design and participants

A cross-sectional design with an additional test–retest measurement for part of the sample was used to assess the psychometric properties of the ICECAP-A. The sample was recruited by a research market agency as part of a larger study aiming to develop ICECAP-A tariffs for the Dutch general population. A sample representative of the Dutch general population, with differences in residential area, educational level, income, and age, was expected to lead to sufficient variations in well-being for this psychometric assessment. An independent medical ethics committee evaluated the study and confirmed it did not fall under the Medical Research Act, waiving the need for ethical approval (METC Leiden-The Hague-Delft, file number N19.119). Hypotheses for the psychometric assessment of the ICECAP-A were registered at AsPredicted (https://aspredicted.org/blind.php?x=sh4dz6) prior to accessing the data, but after data collection. One analysis on convergence and four tests on known-group differences were added later (not preregistered) in order to improve the interpretability of the measurement properties of the ICECAP-A.

### Measurements

#### Demographics

Extracted information on demographics was (1) age in years, (2) current living region or province, (3) gender, (4) highest completed education level with nine categories (ranging from ‘no education’ to ‘university’) that were later transformed to lower, middle, and higher education, (5) employment status with eight categories ranging from ‘unemployed’ to ‘retired’, (6) marital status, and (7) household composition. Furthermore, seven questions likely related to experienced well-being were assessed, namely (1) general happiness on a 4-point scale, (2) general health on a 5-point scale, (3) chronic illness (yes/no) and (4) whether this illness obstructs daily life in any way (yes/no), (5) the amount of visits to a general practitioner or other doctor, (6) if there were any hospital visits in the last 3 months (yes/no), and (7) if there were any hospital stays in the last 3 months (yes/no).

#### ICECAP-A

The ICECAP-A [7] measures five capabilities important to one’s quality of life: (1) stability—the extent to which someone can feel settled and secure; (2) attachment—the extent to which someone can feel love, friendship, and support; (3) autonomy—the extent to which someone can feel independent; (4) achievement—the extent to which someone can experience achievement and success; (5) enjoyment—the extent to which someone can experience enjoyment and pleasure. Four levels are available for each of the five capabilities, ranging from [1] not being able to experience a capability at all to [4] being able to fully experience a capability. The ICECAP-A attempts to capture the extent to which one experiences the freedom to be or carry out what one wishes. ICECAP-A scores were transformed into capability values using tariffs for the Dutch general population (accepted for publication), ranging from 1 (full capability) to 0 (no capability).

#### EQ-5D-5L

The EQ-5D-5L [21] consists of five dimensions (mobility, self-care, usual activities, pain/discomfort, and anxiety/depression) with five levels for each dimension (ranging from “no problems” to “extreme problems/unable to”). Using empirical valuations of the Dutch general public [22] the 3125 possible health states can be transformed to a unique utility score, ranging from 1 (perfect health) to − 0.446 (worse than death) and anchored at 0 (death). The EQ-5D also contains a visual analogue scale which records subject’s self-reported health on a vertical scale ranging from 0 (worst health you can imagine) to 100 (best health you can imagine). For the current study the scale was presented horizontally rather than vertically, to make the question work better on mobile phone.

#### Self-efficacy

Self-reported efficacy was assessed with three questions on a 4-point scale (1 = often, 2 = sometimes, 3 = rarely, 4 = never) regarding the feeling that one’s life is full with possibilities, the feeling to have no control over one’s life, and the feeling that one can do the things one wants to do. The second question was recoded to match the direction of the other two questions, so lower scores reflected higher self-reported efficacy. The sum score (ranging from 3 to 12) was used in construct validity analyses. Additionally, for analyses on known-group differences participants who scored ‘1’ or ‘2’ on all three questions were compared to all other participants.

### Study procedures

Individuals willing to participate were informed about the study and asked for informed consent. They could continue to the questionnaires only after consent was obtained. Information the researchers received from the marketing bureau was anonymous and could not be traced back to individuals. Additionally, a part of the sample who completed the first questionnaire were asked to fill out the same questionnaire after 2 weeks to determine test–retest reliability of the ICECAP-A. At the start of this second assessment participants were asked whether they had experienced a change in health since the previous assessment. Procedures for obtaining informed consent and data handling for the second questionnaire were equal to the first.

### Statistical analyses

#### Reliability

The Intraclass Correlation Coefficient (ICC) was used as index of reliability, since it incorporates both degree of agreement and correlation between measurements. The appropriate approximation of the ICC for the test–retest reliability of the ICECAP-A was calculated following the guideline of Koo and Li [23]. Specifically, a two-way mixed-effects model based on single measurement and aiming for absolute agreement was used to calculate the ICC for ICECAP-A capability values between measurement one and two. An ICC of 0.50–0.75, 0.75–0.90, and greater than 0.90 are considered as moderate, good, and excellent reliability respectively [23].

#### EQ-5D-5L and self-efficacy correlations

Construct validity of the ICECAP-A was evaluated in two ways. First, by investigating correlations of the ICECAP-A with self-efficacy and the EQ-5D-5L. Second, by examining known-group differences. A list of all hypotheses on construct validity can be found in Online Resource 1. Hypothesis 1 (H1) concerned the correlation between ICECAP-A capability values and utility scores of the EQ-5D-5L. While both instruments aim to capture different constructs (i.e., well-being and health), the comparison is relevant to better understand if and when the ICECAP-A can complement generic health measures.

It was expected that the anxiety/depression subscale of the EQ-5D correlated with all subscales of the ICECAP-A (H2–H6), because one of the presumptions of the ICECAP-A is that it is specifically suitable for people with mental health complaints [6]. Higher levels of anxiety/depression were expected to relate to lower scores on the ICECAP-A subscales. Five hypotheses were based on earlier findings that the achievement and, especially, autonomy attributes of the ICECAP-A might relate more strongly to physical health than the other three attributes [24]. Specifically, we expected that having problems concerning mobility, self-care, and usual activities (EQ-5D) would be reflected in lower autonomy scores on the ICECAP-A (H7–H9). Additionally, we expected that reporting problems concerning usual activities and having pain on the EQ-5D would relate negatively to achievement on the ICECAP-A (H10 and H11). Lastly, as chronic pain [25] and leisure time and activities [26] are related to life enjoyment we expected that having problems concerning usual activities and having pain (EQ-5D) would make it more difficult for people to experience enjoyment and pleasure (ICECAP-A; H12 and H13). For all hypotheses we expected a significant medium to high correlation (0.3 < *r* < 0.7) in the direction explained above. The upper boundary to the correlation was set, because we expected the questionnaires and subscales to be related, but also conceptually distinct. Other correlations between the ICECAP-A and EQ-5D subscales were explored, but there were no predetermined expectations.

Lastly, a strong correlation between the ICECAP-A capability values and the self-efficacy sum scores was expected (H14). Spearman rho correlations were used for all hypotheses, since variables were measured at an ordinal level. Multiple testing was accounted for using Holm’s method [27].

#### Known-group differences

Another way of validating the ICECAP-A is to examine its ability to distinguish groups which we know or expect to differ on the construct that the ICECAP-A tries to capture. First, the level of agreement between the two measurements of the ICECAP-A was calculated to give an indication of the stability of repeated scores within participants. Similar to the method used in Gärtner et al. [28] the standard error of measurement (SEM) was used as an indicator of level of agreement. The SEM constitutes the standard deviation of measurement error and can be derived from the error variance of an analysis of variance for repeated measures, including systematic differences: SEM = √(*σ*^2^_time_ + *σ*^2^_error_). After calculating the SEM of the ICECAP-A capability values differences of known groups were calculated. For a hypothesis to be confirmed the differences need to be both statistically significant and greater than the SEM. Known groups were based on self-reported happiness ratings, the visual analogue scale of the EQ-5D, the presence of a chronic illness, the impeding quality of the illness, visits to a general practitioner, visits to a hospital, hospital stays, self-reported self-efficacy, employment status, marital status, and education (H16–26). Details on the hypotheses can be found in Online Resource 1. Hypotheses 16–25 were tested with the Mann–Whitney *U*-test and hypothesis 26 with the Kruskal Wallis test, since the ICECAP-A capability values did not follow a normal distribution. Multiple testing was accounted for using Holm’s method.

#### Sample size

The desired sample size for analyses concerning construct validity including known-group differences was 1000, since then even small correlations (e.g., 0.2) can be determined with high precision (e.g., .06) [29]. For test–retest reliability a sample size of 248 was intended. This would yield a power of 0.9, when the acceptable and expected ICC were estimated to be 0.7 and 0.8 relatively, participants were rated twice and 20% of the participants would not qualify for test–retest analyses [30].

## Results

### Participants

Of the 1002 participants who completed the first assessment, 252 also completed the second assessment. Data from the first assessment were used for investigation of construct validity. Mean completion time of the survey was 13.9 min (SD = 28.0; range 3.8–618.4). Participants who completed the first assessment within five minutes (*N* = 61) were excluded from analyses, due to concerns with regard to the validity of the results. All participants were invited to complete the second assessment, but the assessment was closed when 250 responses were gathered. Data from the second assessment were used for test–retest reliability analysis. On average there were 26.7 days (SD = 2.5) between the first and second assessment. No time limit was set for the second assessment, since it was very brief. However, participants who indicated to have experienced a change in their health (*N* = 44) were excluded from test–retest analysis, since this analysis assumes conditions for participants have remained the same. Finally, data of 941 and 208 participants were used for construct validity and reliability analyses respectively. Characteristics of all included participants are shown in Table 1. Additionally, a comparison of the sample with the Dutch general population can be found in Online Resource 2.

**Table 1:** Means and frequencies of participant characteristics

Variable	Category	Construct validity sample (T1; *N* = 941)	Test–retest sample (T2; *N* = 208)
Age		49.4 (17.1)	56.0 (16.1)
Gender	Female	484 (51.4%)	95 (45.7%)
	Male	455 (48.4%)	113 (54.3%)
	Other	2 (0.2%)	0 (0%)
Education	Primary and/or lower education	192 (20.4%)	52 (25.0%)
	Secondary and/or vocational education	395 (42.0%)	76 (36.5%)
	Higher and/or college education	353 (37.5%)	80 (38.5%)
Marital status	Single	186 (19.8%)	32 (15.4%)
	Living together/married/registered partner	590 (62.7%)	137 (65.9%)
	Relationship	50 (5.3%)	6 (2.9%)
	Divorced	74 (7.9%)	21 (10.1%)
	Widow/widower	33 (3.5%)	9 (4.3%)
	Other	8 (0.9%)	3 (1.4%)
Self-efficacy		5.87 (1.86)	–
ICECAP-A	Capability value	0.88 (0.14)	0.90 (0.13)
EQ-5D-5L	Index scores	0.85 (0.20)	0.86 (0.21)
	Visual analogue scale	76.4 (20.1)	77.3 (19.2)

Values represent mean values with standard deviations in parentheses unless indicated otherwise

### Test–retest reliability

The mean change in ICECAP-A capability value between assessment one and two of the 208 included participants was − .006 (SD = .084). For the 44 excluded participants who reported a change in health since the previous assessment the mean change in ICECAP-A capability values was − .015 (SD = .082). This indicates that the change in ICECAP-A values for these participants was larger than for the included participants who reported no change in health, but still small. The ICC was 0.79 with a 95% confidence interval (CI) of 0.73–0.84, indicating good test–retest reliability. In comparison, the ICC of the EQ-5D was 0.79 (95% CI 0.74–0.84). Reliability estimates and level of agreement for individual items of the ICECAP-A and EQ-5D are presented in Online Resource 3. The results suggest moderate reliability of individual items of the ICECAP-A.

### Construct validity

#### Correlations with the EQ-5D-5L and self-efficacy

Mean capability values of the ICECAP-A and index scores of the EQ-5D-5L can be found in Table 1 and details concerning individual item frequencies of the questionnaires can be found in Online Resource 3. Fourteen hypotheses were tested to investigate the construct validity of the ICECAP-A. Results on all construct validity hypotheses can be found in Table 2 and the correlation matrix between subscales of the ICECAP-A and EQ-5D-5L can be found in Online Resource 4. Mainly, a substantial Spearman correlation between the ICECAP-A capability values and EQ-5D index scores was found (*r* = 0.60). Additionally, the self-efficacy measure showed a strong Spearman correlation of 0.63 with the ICECAP-A capability values, while its correlation with the EQ-5D-5L index scores was less strong (*r* = 0.52). In total, 12 of 14 (86%) were confirmed.

**Table 2:** Results on hypotheses for construct validity

Hypothesis	ICECAP-A scale	Comparator	Spearman’s rho	*p*-value	Confirmed
H1	Capability value	EQ-5D-5L Index score	0.60	< .001	Yes
H2	Stability	Anxiety/depression^a^	0.50	< .001	Yes
H3	Attachment	Anxiety/depression^a^	0.44	< .001	Yes
H4	Autonomy	Anxiety/depression^a^	0.33	< .001	Yes
H5	Achievement	Anxiety/depression^a^	0.38	< .001	Yes
H6	Enjoyment	Anxiety/depression^a^	0.49	< .001	Yes
H7	Autonomy	Mobility^a^	0.25	< .001	No
H8	Autonomy	Self-care^a^	0.27	< .001	No
H9	Autonomy	Usual activities^a^	0.44	< .001	Yes
H10	Achievement	Usual activities^a^	0.48	< .001	Yes
H11	Achievement	Pain/discomfort^a^	0.41	< .001	Yes
H12	Enjoyment	Usual activities^a^	0.37	< .001	Yes
H13	Enjoyment	Pain/discomfort^a^	0.34	< .001	Yes
H14	Capability value	Self-efficacy	0.63	< .001	Yes

^a^Subscale of the EQ-5D-5L

#### Known-group differences

The SEM, based on mean ICECAP-A capability values of the first and second assessment, equalled .0039. This equals 0.39% of the ICECAP-A capability value range, going from 0 to 1. In other words, based on our sample a difference between groups on the ICECAP-A capability value of .0039 or smaller can be attributed to measurement error, while bigger differences are likely due to actual differences between groups. Results on all known-group hypotheses can be found in Table 3. In summary, 10 of 11 (91%) of hypotheses were confirmed. For education, a significant difference was found between groups, but only lower and higher education had a capability value difference that was both significant (*p* = .005) and larger than the SEM, contradicting expectations. The other known-group differences were significant and larger than the SEM, confirming the predetermined hypotheses. Known-group hypotheses were repeated with the EQ-5D-5L index scores to get a better understanding of the difference between the EQ-5D-5L and the ICECAP-A. Results on these analyses can be found in Online Resource 5. Both questionnaires performed similarly in distinguishing known groups. When looking at the size of the median difference between tested known groups in relation to the SEM the EQ-5D-5L might distinguish groups based on hospital visits and hospital stays more clearly than the ICECAP-A, while the ICECAP-A might be especially good in distinguishing groups based on happiness, overall health (based on EQ-5D-5L VAS scores), self-efficacy, employment, and relationship status.

**Table 3:** Results on hypotheses for known-group differences

Hypothesis	Known group	*N*	Mean rank score	Median	Range	*p*-value	Confirmed
H16	Happy	800	515	0.9428	0.0–1.0	< .001	Yes
	Unhappy	141	219	0.7562	0.3–1.0		
H17	VAS ≥ 65	714	540	0.9448	0.4–1.0	< .001	Yes
	VAS < 65	227	255	0.7879	0.0–1.0		
H18	No illness	562	564	0.9495	0.4–1.0	< .001	Yes
	Illness present	379	334	0.8546	0.0–1.0		
H19^a^	Non-obstructing illness	51	255	0.9226	0.5–1.0	< .001	Yes
	Obstructing illness	328	180	0.8312	0.0–1.0		
H20	No hospital visit	588	511	0.9375	0.2–1.0	< .001	Yes
	Hospital visit	353	405	0.9149	0.0–1.0		
H21	No hospital stay	860	477	0.9305	0.0–1.0	= .017	Yes
	Hospital stay	81	402	0.9149	0.4–1.0		
H22	No GP visit	383	549	0.9475	0.2–1.0	< .001	Yes
	GP visit	558	417	0.9149	0.0–1.0		
H23	High self-efficacy	415	601	0.9565	0.5–1.0	< .001	Yes
	Low self-efficacy	526	368	0.8790	0.0–1.0		
H24	Employed	811	501	0.9375	0.0–1.0	< .001	Yes
	Unemployed/occupational disability	130	283	0.8144	0.2–1.0		
H25	Relationship	640	504	0.9375	0.3–1.0	< .001	Yes
	No relationship	301	401	0.9070	0.0–1.0		
H26^b^	Higher education	353	NA	0.9339	0.2–1.0	.021	No
	Medium education	395		0.9339	0.3–1.0		
	Lower education	192		0.9149	0.0–1.0		

*GP* general practitioner; *VAS* visual analogue scale of the EQ-5D-5L

^a^This question was only applicable to 379 participants who indicated to have a chronic illness

^b^One subject is missing from this analysis since the response to this question was not interpretable

## Discussion

The aim of this study was to assess the psychometric properties of the ICECAP-A in a large sample representative of the general Dutch population. The instrument showed good test–retest reliability with an ICC of 0.79. Good construct validity was found based on correlations with the EQ-5D-5L and a measure of self-efficacy, with 12 of 14 hypotheses (86%) being confirmed. Similarly, the ICECAP-A showed adequate construct validity by being able to differentiate between known groups, with 10 of 11 hypotheses (91%) being confirmed.

In general, correlations between the ICECAP-A and EQ-5D-5L were moderate to strong. This result suggests that while there is considerable overlap between the two instruments, there may be a difference in the underlying measured constructs. Interestingly, the correlation between the autonomy subscale of the ICECAP-A and the EQ-5D subscales self-care and mobility was poor (smaller than 0.3, though still significant). This is surprising given that difficulties with moving and taking care of oneself imply that help from others is needed. It might be that such difficulties can be overcome without help from others, through the use of (walking) aids or extra effort, or that aspects of autonomy not related to physical capabilities, such as being able to make choices, explain the variance on the autonomy item better. Another explanation is that a ceiling effect on the EQ-5D dampened the correlation. Indeed, 70% and 91% of the participants reported the highest level of mobility (i.e., ‘no problems with walking’) and self-care (i.e., ‘no problems with washing and getting dressed’), respectively. For the autonomy subscale of the ICECAP-A considerably less participants (48%) reported the highest level (i.e., ‘able to be completely independent’). Overall 33% of the participants reported the maximum score on the EQ-5D, whereas 14% did so for the ICECAP-A. This suggests that the ICECAP-A, compared to the EQ-5D, might have more room to detect subtle changes in quality of life. This heightened sensitivity has been established in other populations [5, 6].

Contrary to our hypothesis, the difference in capability value did not exceed the SEM while also being significant for all three educational groups. Only the comparison between higher and lower educational groups fulfilled both criteria. The hypothesis was based on earlier research indicating that the EQ-5D could discriminate similar groups [31], but an additional analysis suggested that the EQ-5D, compared to the ICECAP-A, performed roughly equal in discriminating the three educational groups in the current sample. Regarding other known-group differences, the EQ-5D-5L seemed to distinguish groups more clearly than the ICECAP-A when groups were based on hospital visits and hospital stays. This seems further evidence that the EQ-5D-5L puts more emphasis on health, while the ICECAP-A has a broader focus. Indeed, the ICECAP-A distinguished groups more clearly when groups were based on concepts related to general well-being, such as happiness, relationship status, and self-efficacy. These results are in line with earlier research suggesting that the ICECAP-A correlated positively with feelings of happiness and freedom [9]. Moreover, the self-efficacy measure correlated strongly with the ICECAP-A capability value, indicating they measured overlapping concepts. The substantial correlation should not be surprising, since self-efficacy is defined as an individual’s belief about their own capabilities and mastery over their life [32] which seems very similar to the construct of the ICECAP-A as described by the developers [7].

### Previous research and implications

Regarding test–retest reliability, similar results were established in a previous studies. A slightly higher ICC of 0.86 for the ICECAP-A capability values was found in a sample from the Danish population [14] and an ICC of 0.72 was found in a general UK sample [13]. In this UK study, reliability of the ICECAP-A was found to be lower than the EQ-5D, which might be explained in part by the inherent property of capabilities being harder to objectify than health. Indeed, the current study also showed a lower test–retest reliability of individual items of the ICECAP-A compared to those of the EQ-5D. However, no difference between the ICC estimates of the ICECAP-A capability values and EQ-5D index scores was found.

The same research team also found comparable results regarding validity [9]. In a sample of 418 participants representative of the general UK population 97 hypotheses were formed regarding construct validity of which 67 (69%) were confirmed. It must be noted that multiple comparisons were not accounted for, which likely increased the amount of significant findings. Nevertheless, the authors stated that while their research does not indicate definitive validity of the ICECAP-A, it does show potential in capturing intervention benefits because of its ability to identify relevant differences between groups. This statement is solidified in other studies. For example, in a substance dependence sample Goranitis et al. [5] found that the ICECAP-A has stronger correlations than the EQ-5D with concepts that are often important objectives of interventions, such as social support, functioning, and well-being. Additionally, compared to the EQ-5D the ICECAP-A was found to be more sensitive to change, which has been reproduced in a sample with depression [6], and advocates its use in samples suffering from chronic or mental disorders. However, this does not mean that capability instruments like the ICECAP-A should replace health questionnaires like the EQ-5D. Combining previous findings with that of the current study suggests that the ICECAP-A will perform especially well in contexts outside of the traditional health intervention model, while generic health questionnaires will do better when health is the outcome of interest. Indeed, previous studies [11, 24] and the NICE social care guidelines [33] suggest that the two instruments assess different constructs and can effectively complement each other. The Dutch guidelines for conducting economic evaluations in healthcare also specify that the ICECAP should be added when interventions aim to improve not only health gain, but well-being in terms of living situation, autonomy, and social interaction as well [34].

### Strength, limitations, and future directions

A strength of this psychometric evaluation was that the study was preregistered to ensure reliable hypotheses testing. Secondly, appropriate statistical choices were made such as using a suitable ICC, correcting for multiple testing, and examining both the significance and size of correlations and differences. Thirdly, a large sample representative of the general Dutch population was used. Quotations based on age, gender, and income were used during recruitment, resulting in a heterogeneous sample regarding health, well-being, happiness, and education level, and a good starting point for assessing psychometric properties. Future studies exploring the responsiveness of the ICECAP-A should consider more specific populations.

Admittedly, some limitations can be indicated. First, the ICECAP-A was administered online only so results do not necessarily generalize to a paper–pencil version of the questionnaire. However, there are no reasons to expect a difference between the two methods and earlier work confirms this for the EQ-5D-5L [35]. Second, for construct validity the ICECAP-A was compared to the EQ-5D-5L and a measure of self-efficacy. Including other quality of life, health or capability instruments, and assessment of discriminative validity might have led to an enhanced understanding of the psychometric properties of the ICECAP-A. Nevertheless, the current analyses add to the understanding of the ICECAP-A construct and its added value to health-related quality of life measures. Third, regarding test–retest reliability, there was on average 26.7 days between assessment one and two which may have introduced recall bias. Lastly, changes in well-being at the second assessment were assessed by asking participants whether they had experienced a change in health since the previous assessment rather than also informing on changes in well-being. While there was a larger decline in ICECAP-A capability values in the group who reported a change in health since the first assessment, the change was still small, questioning the appropriateness of this check of changes in well-being.

## Conclusion

Adequate psychometric properties of the ICECAP-A are vital to be able to reliably use the instrument. The present study adds to the established literature on the psychometric properties of the ICECAP-A by showing good test–retest reliability and construct validity in a large Dutch sample. The instrument demonstrates both overlap and differences with the EQ-5D-5L, indicating that the ICECAP-A might measure a distinct concept, closely related to well-being and self-efficacy, that is influenced by health status. Consequently, the ICECAP-A can complement other generic health questionnaires when attempting to capture the benefits of interventions outside the traditional health intervention model.

## Supplementary information

Below is the link to the electronic supplementary material.

Supplementary material 1 (PDF 323 kb)

## Data Availability

Data are available from the authors upon request.

## Code availability

SPSS syntax is available from the authors upon request.
