# Supplementary material for: The ICECAP-A instrument for capabilities: assessment of construct validity and test–retest reliability in a general Dutch population
Source: Qual Life Res. 2021 Aug 31;31(3):687–96. doi: 10.1007/s11136-021-02980-5 (PMC8921020; doi:10.1007/s11136-021-02980-5)
Supplement: Supplementary file 1 — Supplementary material 1 (PDF 323 kb) [file 11136_2021_2980_MOESM1_ESM.pdf]

Online Resources for 'The ICECAP-A instrument for capabilities: assessment of construct validity and test-retest reliability in a general Dutch population'

Pieter J. Rohrbach, Alexandra E. Dingemans, Brigitte A. Essers, Eric F. Van Furth, Philip Spinhoven, Catharina G. M. Groothuis-Oudshoorn, Janine A. Van Til, M. Elske Van den Akker-Van Marle*

*Corresponding author, e-mail: m.e.van_den_akker-van_marle@lumc.nl

Online Resource 1. List of all predetermined construct validity hypotheses

Construct validity hypotheses
For all hypotheses we expected a significant medium to high correlation ($0.3 < r < 0.7$) in the direction explained in the article text.

- H1: ICECAP-A capability values and the EQ-5D utility scores;
- H2: ICECAP-A stability subscale and EQ-5D anxiety/depression subscale;
- H3: ICECAP-A attachment subscale and EQ-5D anxiety/depression subscale;
- H4: ICECAP-A autonomy subscale and EQ-5D anxiety/depression subscale;
- H5: ICECAP-A achievement subscale and EQ-5D anxiety/depression subscale;
- H6: ICECAP-A enjoyment subscale and EQ-5D anxiety/depression subscale;
- H7: ICECAP-A autonomy subscale and EQ-5D mobility subscale;
- H8: ICECAP-A autonomy subscale and EQ-5D self-care subscale;
- H9: ICECAP-A autonomy subscale and EQ-5D usual activities subscale;
- H10: ICECAP-A achievement subscale and EQ-5D usual activities subscale;
- H11: ICECAP-A achievement subscale and EQ-5D pain subscale;
- H12: ICECAP-A enjoyment subscale and EQ-5D usual activities subscale;
- H13: ICECAP-A enjoyment subscale and EQ-5D pain subscale.

A hypothesis was added later (not preregistered) to improve the interpretability of the ICECAP-A measurement properties. A strong correlation was expected between the ICECAP-A capability values and a 3-item measure of self-efficacy.

- H14: ICECAP-A capability values and self-efficacy.

Known-group hypotheses
For a hypothesis to be confirmed the differences need to be both statistically significant and greater than the SEM. The SEM can be derived from the error variance of an analysis of variance for repeated measures, including systematic differences: SEM = $\sqrt{(\sigma^2_{time} + \sigma^2_{error})}$. Note that hypotheses 19-22 were added later (not preregistered).

- H16: Higher ICECAP-A scores for participants who indicated to be very happy or moderately happy as opposed to participants who indicated to be not very happy or unhappy;
- H17: Higher ICECAP-A scores for participants who indicated to be closer to the best health they could imagine as indicated by the visual analogue scale of the EQ-5D (score between 66 and 100) as opposed to participants indicating being further away from the best health they could imagine (score between 0 and 65). The cutoff score on the visual analogue scale of the EQ-5D for this hypothesis was based on the average and standard deviation of general populations in earlier research, which mostly had a mean of around 80 and standard deviation of around 15 (on a scale of 0 to 100);
- H18: Higher ICECAP-A scores for participants who indicated to have a long-lasting illness as opposed to participants who indicated not to have one;
- H19: Lower ICECAP-A scores for participants who indicated that the long-lasting illness (as reported in H16) obstructed daily life as opposed to participants who indicated that this was not the case.
- H20: Lower ICECAP-A scores for participants who indicated to have been to the hospital in the last three months to visit a doctor as opposed to participants who have not been to the hospital in this period.
- H21: Lower ICECAP-A scores for participants who indicated to have had to stay (spend at least one night) in the hospital in the last three months as opposed to participants for whom this was not the case.
- H22: Lower ICECAP-A scores for participants who indicated to have had at least one visit to the general practitioner in the last three months as opposed to participants for whom this was not the case.

- H23: Higher ICECAP-A scores for people who indicated more self-efficacy in their lives. Self-reported efficacy was assessed with three questions on a 4-point scale (1=often, 2=sometimes, 3=rarely, 4=never) regarding the feeling that one's life is full with possibilities, the feeling to have no control over one's life, and the feeling that one can do the things one wants to do. After recoding the second question, lower scores reflected higher self-reported efficacy. The compared groups were participants who indicated 'often' or 'sometimes' on all three questions versus all other participants;
- H24: Lower ICECAP-A scores for participants who indicated to be unemployed or have an occupational disability as opposed to all other participants;
- H25: Higher ICECAP-A scores for participants who indicated to be in a relationship as opposed to participants who indicated to fall under the category single, divorced, widow or other;
- H26: Higher ICECAP-A scores for participants who indicated to have enjoyed higher education. Three groups were made based on previous research with the EQ-5D [1], being primary and/or lower education, secondary and/or vocational education and higher and/or college education.

References

[1] Janssen, M. F., Pickard, A. S., Golicky, D., Gudex, C., Niewada, M., Scalone, L., Swinburn, P., Busschbach, J. (2012). Measurement properties of the EQ-5D-5L compared to the EQ-5D-3L across eight patient groups: a multi-country study. *Quality of Life Research*, *22*(7)**,** 1717-1727. https://doi.org/10.1007/s11136-012-0322-4

Online Resource 2. Comparison of sample with Dutch population on target variables

Variable	Category	Construct validity sample (T1; *N*=941)	Test-retest sample (T2; *N*=208)	Dutch population[a]
Age groups	18-24	9.4%	5.8%	11.0%
	25-34	15.0%	9.1%	16.0%
	35-44	14.5%	7.7%	15.0%
	45-54	18.4%	15.9%	18.0%
	55-64	17.6%	21.2%	17.0%
	65-74	20.4%	32.7%	14.0%
	75-99	4.8%	7.7%	10.0%
Gender	Female	51.4%	45.7%	49.11%
	Male	48.4%	54.3%	50.89%
	Other	0.2%	0.0%	Unknown
Region	Groningen	3.5%	5.3%	3.0%
	Friesland	3.4%	5.3%	4.0%
	Drenthe	3.1%	5.8%	3.0%
	Overijssel	8.4%	6.3%	7.0%
	Gelderland	11.6%	11.1%	12.0%
	Flevoland	2.2%	4.8%	2.0%
	Utrecht	8.6%	7.7%	7.0%
	Noord-Holland	16.7%	12.0%	16.0%
	Zuid-Holland	18.5%	16.3%	21.0%
	Zeeland	2.2%	3.4%	2.0%
	Noord-Brabant	14.0%	14.9%	15.0%
	Limburg	7.8%	7.2%	7.0%
Income	<€11.500	5.4%	3.4%	5.0%
	€11.500 - €30.000	28.6%	34.6%	26.0%
	€30.000 - €36.000	10.4%	11.1%	9.0%
	€36.000 - €60.500	31.0%	25.0%	33.0%
	>€60.500	20.7%	22.6%	27.0%
	Rather not tell	3.8%	3.4%	Not applicable
Education	High	37.5%	38.5%	34.2%[b]
	Middle	42.0%	36.5%	37.8%[b]
	Low	20.4%	25.0%	26.3%[b]
	Missing/Unknown	0.1%	0.0%	1.6%[b]

[a] Numbers are based on the latest numbers known to the market research agency unless indicated otherwise.
[b] Numbers are based on 2020 education statistics of the Netherlands' Central Bureau of Statistics.
Note. The selection of a sample representative of the Dutch population was based on the age, gender, region and income variables. Other variables such as education, religion and ethnicity were not considered.

Online Resource 3. Individual item details of the ICECAP-A and EQ-5D-5L

Gwet's AC2 [1] was preferred over the intraclass correlation coefficient as test-retest reliability parameter for the individual items of the ICECAP-A and EQ-5D-5L as it is appropriate for ordinal outcomes and skewed data [2, 3]. A Gwet's AC2 of 0.4-0.6, 0.6-0.8 and greater than 0.8 was considered as moderate, good and excellent reliability respectively.

3a. ICECAP-A individual item frequencies (%) and reliability for the study sample

Capability	Level 1[a]	Level 2[a]	Level 3[a]	Level 4[a]	Mean (*SD*)[a]	Gwet's AC2 [95% CI][b]	Level of agreement[b]
Stability	12 (1.3)	108 (11.5)	425 (45.2)	396 (42.1)	3.3 (0.7)	0.64 [0.54; 0.73]	70.7%
Attachment	8 (0.9)	144 (15.3)	382 (40.6)	407 (43.3)	3.3 (0.7)	0.59 [0.49; 0.69]	67.3%
Autonomy	16 (1.7)	79 (8.4)	395 (42.0)	451 (47.9)	3.4 (0.7)	0.62 [0.52; 0.71]	68.8%
Achievement	31 (3.3)	191 (20.3)	456 (48.5)	263 (27.9)	3.0 (0.8)	0.51 [0.39; 0.62]	61.1%
Enjoyment	16 (1.7)	148 (15.7)	422 (44.8)	355 (37.7)	3.2 (0.8)	0.58 [0.48; 0.69]	66.8%

Note. Values represent frequencies with percentages in parentheses unless indicated otherwise. Level 1 corresponds to 'not being able to experience a capability at all' and level 4 to 'being able to fully experience a capability'.
SD = Standard deviation.
[a] Values are based on the total study sample (*N*=941)
[b] Values are based on the test-retest sample (*N*=208)

3b. EQ-5D-5L individual item frequencies (%) and reliability for the study sample

Domain	Level 1[a]	Level 2[a]	Level 3[a]	Level 4[a]	Level 5[a]	Mean (*SD*)[a]	Gwet's AC2 [95% CI][b]	Level of agreement[b]
Mobility	7 (0.7)	33 (3.5)	66 (7.0)	180 (19.1)	655 (69.6)	4.5 (0.8)	0.75 [0.68; 0.83]	78.4%
Self-care	9 (1.0)	4 (0.4)	18 (1.9)	58 (6.2)	852 (90.5)	4.9 (0.6)	0.92 [0.87; 0.96]	91.8%
Usual activities	12 (1.3)	30 (3.2)	94 (10.0)	201 (21.4)	604 (64.2)	4.4 (0.9)	0.78 [0.71; 0.84]	80.3%
Pain/ discomfort	8 (0.9)	50 (5.3)	131 (13.9)	325 (34.5)	427 (45.4)	4.2 (0.9)	0.59 [0.5; 0.69]	65.9%
Anxiety/ depression	7 (0.7)	28 (3.0)	81 (8.6)	196 (20.8)	629 (66.8)	4.5 (0.8)	0.74 [0.66; 0.81]	76.4%

Note. Values represent frequencies with percentages in parentheses unless indicated otherwise. Level 1 corresponds to 'extreme problems/unable to' and level 5 to 'no problems'.
SD = Standard deviation.
[a] Values are based on the total study sample (*N*=941)
[b] Values are based on the test-retest sample (*N*=208)

References

[1] Gwet, K. L. (2008). Computing inter-rater reliability and its variance in the presence of high agreement. *British Journal of Mathematical and Statistical Psychology, 61*(Pt 1), 29–48. https://doi.org/10.1348/000711006X126600

[2] Tran, D., Dolgun, A., & Demirhan, H. (2020). Weighted inter-rater agreement measures for ordinal outcomes. *Communications in Statistics-Simulation and Computation, 49*(4), 989–1003. https://doi.org/10.1080/03610918.2018.1490428

[3] Long, D., Polinder, S., Bonsel, G. J., & Haagsma, J. A. (2021). Test–retest reliability of the EQ-5D-5L and the reworded QOLIBRI-OS in the general population of Italy, the Netherlands, and the United Kingdom. *Quality of Life Research*, 1-11. https://doi.org/10.1007/s11136-021-02893-3

Online Resource 4. Correlation matrix of ICECAP-A and EQ-5D-5L index scores and subscales

	EQ-5D index score	Mobility	Self-care	Usual activities	Pain/discomfort	Anxiety/depression	Visual analogue scale
ICECAP capability score	0.60[a]	0.29	0.28	0.50	0.41	0.57	0.58
Stability	0.44	0.13	0.15	0.32	0.30	0.50[a]	0.41
Attachment	0.33	0.11	0.15	0.23	0.16	0.44[a]	0.36
Autonomy	0.45	0.25[a]	0.27[a]	0.44[a]	0.32	0.33[a]	0.42
Achievement	0.53	0.33	0.26	0.48[a]	0.41[a]	0.38[a]	0.51
Enjoyment	0.47	0.24	0.18	0.37[a]	0.34[a]	0.49[a]	0.46

Note. All presented correlations are significant with p-value < .001.

[a] Correlation for which predetermined hypotheses were composed.

Online Resource 5. Results on hypotheses for known-group differences repeated for the EQ-5D-5L

Hypothesis	Known group	*N*	Mean rank score	Median	Range	*p*-value	Confirmed
H16	Happy	800	512	0.9340	-0.4;1.0	<.001	Yes
	Unhappy	141	236	0.7540	-0.1;1.0		
H17	VAS >= 65	714	558	0.9340	0.0;1.0	<.001	Yes
	VAS < 65	227	197	0.7260	-0.4;1.0		
H18	No illness	562	601	0.9650	0.3;1.0	<.001	Yes
	Illness present	379	278	0.7900	-0.4;1.0		
H19[a]	Non-obstructing illness	51	281	0.9340	0.3;1.0	<.001	Yes
	Obstructing illness	328	176	0.7680	-0.4;1.0		
H20	No hospital visit	588	542	0.9340	-0.1;1.0	<.001	Yes
	Hospital visit	353	352	0.8340	-0.4;1.0		
H21	No hospital stay	860	485	0.9300	-0.4;1.0	<.001	Yes
	Hospital stay	81	319	0.8250	0.1;1.0		
H22	No GP visit	383	582	0.9650	0.0;1.0	<.001	Yes
	GP visit	558	395	0.8640	-0.4;1.0		
H23	High self-efficacy	415	583	0.9610	0.2;1.0	<.001	Yes
	Low self-efficacy	526	382	0.8640	-0.4;1.0		
H24	Employed	811	504	0.9340	-0.4;1.0	<.001	Yes
	Unemployed/ occupational disability	130	265	0.7640	0.0;1.0		
H25	Relationship	640	486	0.9300	0.0;1.0	=.011	Yes
	No relationship	301	439	0.8950	-0.4;1.0		
H26[b]	Higher education	353	NA	0.9340	0.1;1.0	=.002	No
	Medium education	395		0.9300	-0.1;1.0		
	Lower education	192		0.8750	-0.4;1.0		

The standard error of measurement (SEM) of the EQ-5D-5L was calculated to be .0133.
GP = General practitioner; VAS = Visual analogue scale of the EQ-5D-5L.
[a] This question was only applicable to 379 participants who indicated to have a chronic illness.
[b] One subject is missing from this analysis since the response to this question was not interpretable.
